# Case of Colorectal Cancer After Cold Snare Polypectomy Successfully Salvaged on Endoscopic Submucosal Dissection

**DOI:** 10.7759/cureus.11182

**Published:** 2020-10-26

**Authors:** Atsushi Katagiri, Kazuya Inoki, Kenichi Konda, Fuyuhiko Yamamura, Hitoshi Yoshida

**Affiliations:** 1 Department of Medicine, Division of Gastroenterology, Showa University School of Medicine, Tokyo, JPN

**Keywords:** colorectal cancer, cold snare polypectomy, residual cancer, endoscopic submucosal dissection, additional therapy

## Abstract

Cold snare polypectomy is a well-established method for the resection of colorectal polyps measuring less than 10 mm in size. It may be performed in patients with early colorectal cancers because of the difficulty of endoscopic diagnosis. However, the therapeutic effect of cold snare polypectomy on cancers is unknown, and the need for appropriate surveillance and additional treatment for these patients remains to be fulfilled. Endoscopic submucosal dissection has been reported as a safe and effective method for treating recurrent or residual colorectal neoplasia following hot endoscopic mucosal resection.

This report is of a case of a colorectal tumor measuring 8 mm that was treated using cold snare polypectomy and resulted in residual cancer. Endoscopic submucosal dissection was selected as salvage treatment for the residual lesion, and histopathological examination revealed free horizontal and vertical margins.

We believe that performing endoscopic submucosal dissection at the site of the cancer resected with cold snare polypectomy ensured that there was no residual cancer left. It may be hypothesized that endoscopic submucosal dissection could evolve as the treatment of choice for patients with colorectal cancer after cold snare polypectomy.

## Introduction

Colonoscopic removal of a neoplastic polyp is the most effective therapeutic measure for colorectal cancer prevention [1,2]. Cold snare polypectomy (CSP) is a well-established method for the resection of colorectal polyps measuring less than 10 mm, as it is associated with a low frequency of post-procedural bleeding and perforation [3,4]. Although the proportion of malignancy in colorectal neoplastic lesions measuring less than 10 mm is considered very low, small lesions with advanced histology have been reported [5].

Regardless of the high diagnostic accuracy of magnifying colonoscopy in colorectal neoplasia, there are early cancers that are difficult to diagnose endoscopically [6,7]. As a result, CSP may potentially be applied to early colorectal cancers for which it is not ordinarily indicated. As a characteristic of CSP, some reports have demonstrated that it is difficult to evaluate the pathological margin of a specimen resected using CSP, which is the gold standard for determining curative resection [8,9]. Accordingly, it is difficult to pathologically verify complete resection in these cancers after CSP. Thus, the therapeutic effect of CSP on colorectal cancers is unknown at present. Hence, there is a lack of consensus on the appropriate further management for patients with early colorectal cancer resected using CSP.

With respect to treating residual lesions, endoscopic submucosal dissection (ESD) has been reported as a safe and effective method for treating recurrent or residual colorectal neoplasia following hot endoscopic resection [10]. Colorectal ESD may be an effective salvage treatment for cancers resected using CSP, which is considered an inadequate therapy for early colorectal cancer.

We report a case of colorectal cancer after CSP that was successfully managed using ESD.

## Case presentation

**Figure 1:**
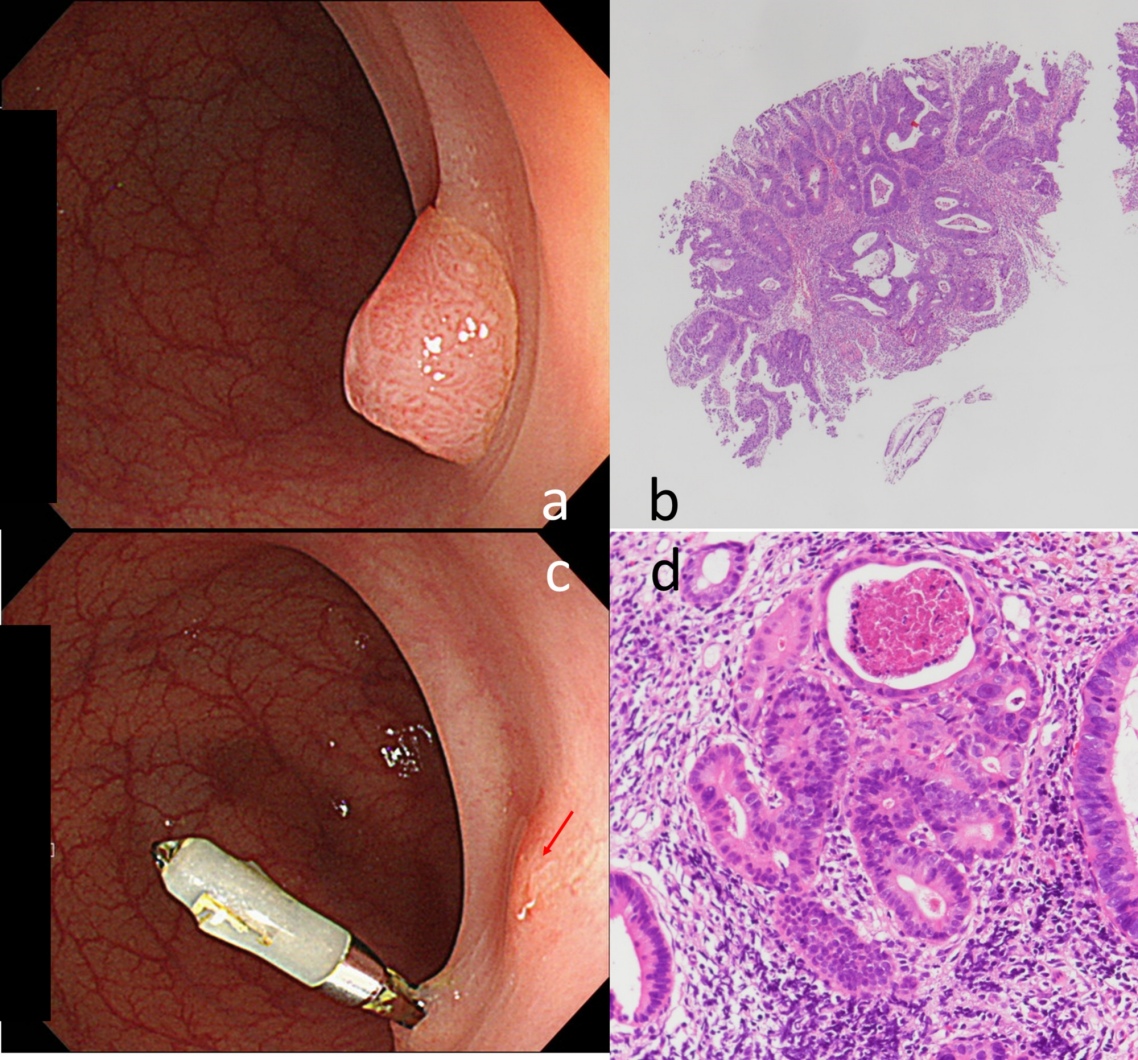
Endoscopic and pathological findings of pretreatment and recurrent lesions 1a: Colonoscopic image showing a polypoidal lesion in the sigmoid colon (before cold snare polypectomy). 1b: Histopathological image of the specimen resected using cold snare polypectomy (CSP) showing well-to-moderately differentiated adenocarcinoma (Hematoxylin and eosin [H&E]). The vertical margin status is unclear. 1c: Post CSP colonoscopy showing residual lesion recognized as a submucosal tumor (red arrow). Note the hemostatic clip from the previous procedure. 1d: Histopathology of the endoscopic biopsy showing moderately differentiated adenocarcinoma (H&E). This is suggestive of residual carcinoma.

A 76-year-old woman underwent colonoscopy at a medical facility, and an 8 mm pedunculated lesion was found in her sigmoid colon (Figure 1a). The lesion was resected using CSP and a residual lesion was not found at the time of resection. Histopathology revealed a well-to-moderately differentiated adenocarcinoma with unknown vertical and horizontal margin statuses (Figure 1b). A follow-up colonoscopy performed approximately six months later revealed a submucosal tumor (SMT) measuring 2 mm near the initial resection site (Figure 1c). Biopsy confirmed moderately differentiated adenocarcinoma, and this lesion was diagnosed as a residual tumor (Figure 1d).

**Figure 2:**
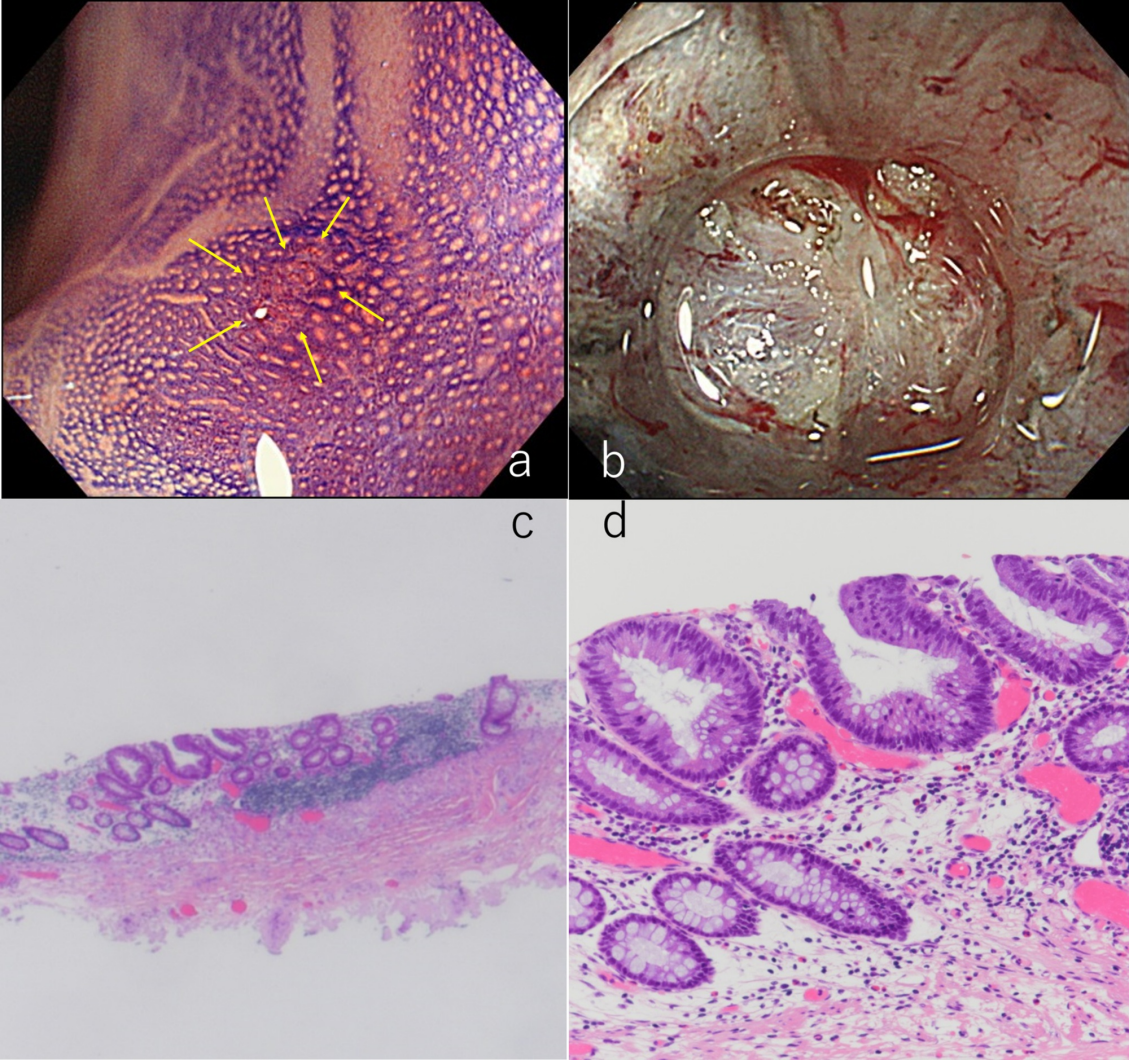
Magnifying colonoscopy for the residual lesion after biopsy and endoscopic submucosal dissection for this lesion 2a: Chromoendoscopy with magnification showing a residual lesion with an irregular pit pattern (yellow arrows). 2b: Submucosal fibrosis due to the previous treatment noted during the endoscopic submucosal dissection (ESD) procedure. 2c: Loupe magnification image of the specimen resected en bloc using ESD (H&E). The vertical and horizontal margins are free of tumor. 2d: Photomicrograph of resected specimen following ESD showing low-grade neoplasia (H&E).

Magnifying colonoscopy was then performed eight months after the initial colonoscopy. It showed disappearance of the SMT and a flat 1-mm lesion with an irregular pit at the same site (Figure 2a). The patient requested endoscopic resection when the patient was presented with endoscopic resection and surgical resection as salvage treatment for this residual lesion. ESD was selected as the salvage treatment for this residual lesion for both residual tumor excision and proving that there was no other residual cancer in the submucosa. Submucosal fibrosis due to the previous treatment (Figure 2b) did not pose any complications during ESD. Histopathological examination revealed low-grade dysplasia with free horizontal and vertical margins (Figure 2c, 2d).

## Discussion

To our knowledge, this is the first report of a successful salvage using ESD for residual colorectal cancer after CSP. Colonoscopy and the removal of neoplastic polyps are very effective for colorectal cancer prevention [1,2], and CSP is a well-established method, owing to its safety, for the resection of colorectal polyps measuring less than 10 mm [3,4]. The adaptation limit of lesion size in CSP is because of its en-block resection ability [3,4] and the low frequency of carcinomas. Although the proportion of colorectal cancer in colorectal neoplastic lesions measuring less than 10 mm is thought to be very low, there are lesions with an advanced histology regardless of their small size [5]. Dye-assisted magnifying colonoscopy and image enhancement have high diagnostic accuracy for not only differentiating neoplastic and non-neoplastic lesions but also for the prediction of invasion depth in submucosal cancers [6,7]. However, it is not always possible to perform magnifying colonoscopy in daily medical care, and there is a possibility of CSP being performed for colorectal cancers owing to the difficulty in diagnosing them endoscopically. Moreover, it is difficult to evaluate the pathological margin of a specimen resected using CSP, which is the gold standard for determining curative resection [8,9]. As far as we know, there are no reports on the effect of CSP on colorectal cancer; therefore, the therapeutic effect of CSP on colorectal cancers is unknown. The need for appropriate surveillance remains to be fulfilled and how these patients should be additionally treated is also unclear. This case highlights the importance of meticulous follow-up colonoscopy for malignant polyp after CSP. In this case, the residual lesion showed SMT-like findings on endoscopy. Although SMT-like morphology is associated with T1b cancer, we did not consider this lesion to be T1b cancer before CSP. The reason for our assumption is that the pathological findings of the CSP specimen did not recognize desmoplastic reaction as a characteristic finding of T1b cancer. In addition, there is no specific literature on the recurrence pattern of colorectal cancer after CSP. Attention should thus be paid to SMT-like findings around the scar after excision. The appropriate additional therapy for colorectal cancer resected using CSP is currently unknown, although ESD is safe and effective for the treatment of recurrent or residual colorectal tumors using hot endoscopic resection [10]. ESD allows for accurate excision of the submucosal layer under visualization and also enables the effective treatment of cases with pre-existing fibrosis such as recurrence after endoscopic resection. In our case, endoscopic mucosal resection was considered difficult, since fibrosis of the submucosal layer due to previous CSP was assumed. Further, we thought that there was a residual tumor based on the biopsy results, and the absence of cancer in the submucosal layer had to be confirmed; hence, we chose ESD as the appropriate salvage therapy for this lesion. The difference between the histological findings of the biopsy and the ESD specimen can be explained by the possibility that only the adenomatous component of the lesion was left. It could also be possible that another lesion near the ESD site was accidentally resected simultaneously or exfoliated tumor cells implanted to the mucosal defect after CSP. In any case, we believe that performing an ESD ensured that there was no residual cancer left after CSP.

## Conclusions

We report a case of residual colorectal cancer after CSP that was successfully managed using ESD. It may be hypothesized that ESD could evolve as a treatment for such cases. However, the therapeutic efficacy of ESD as an additional treatment for these cases is currently unknown in the given clinical context and further studies are required to validate the same.
